# Inhibition of the Binding between RGS2 and β-Tubulin Interferes with Spindle Formation and Chromosome Segregation during Mouse Oocyte Maturation *In Vitro*

**DOI:** 10.1371/journal.pone.0159535

**Published:** 2016-07-27

**Authors:** Man-Xi Jiang, Yan Shi, Zhao-Gui Sun, Zhi Zhang, Yan Zhu

**Affiliations:** 1 Guangdong No.2 Provincial People’s Hospital, Guangzhou 510317, China; 2 NPFPC Key Lab of Contraceptives and Devices, Shanghai Institute of Planned Parenthood Research, Shanghai 200032, China; Institut de Génétique et Développement de Rennes, FRANCE

## Abstract

RGS2 is a negative regulator of G protein signaling that contains a GTPase-activating domain and a β-tubulin binding region. This study aimed to determine the localization and function of RGS2 during mouse oocyte maturation in vitro. Immunofluorescent staining revealed that RGS2 was widely expressed in the cytoplasm with a greater abundance on both meiotic spindles and first/second polar bodies from the fully-grown germinal vesicle (GV) stage to the MII stages. Co-expression of RGS2 and β-tubulin could also be detected in the spindle and polar body of mouse oocytes at the MI, AI, and MII stages. Inhibition of the binding site between RGS2 and β-tubulin was accomplished by injecting anti-RGS2 antibody into GV-stage oocytes, which could result in oocytes arrest at the MI or AI stage during in vitro maturation, but it did not affect germinal vesicle breakdown. Moreover, injecting anti-RGS2 antibody into oocytes resulted in a significant reduction in the rate of first polar body extrusion and abnormal spindle formation. Additionally, levels of phosphorylated MEK1/2 were significantly reduced in anti-RGS2 antibody injected oocytes compared with control oocytes. These findings suggest that RGS2 might play a critical role in mouse oocyte meiotic maturation by affecting β-tubulin polymerization and chromosome segregation.

## Introduction

In mammals, the ovarian follicle consists of an oocyte and one or more layers of granulosa cells, which represent the functional unit of the ovary[1]. An oocyte within the follicle is originally immature and arrested in the first meiotic prophase (prophase I); arrest is maintained by the somatic cell compartment of the follicles[2,3]. An oocyte arrested at prophase I has an intact nuclear envelope or germinal vesicle (GV), and germinal vesicle break down (GVBD) is the first visible event that indicates the resumption of meiosis. After meiosis resumption, the first meiotic spindle forms in the center of the oocyte and then migrates to the cortex at the end of metaphase I (MI)[4,5], prior to cytokinesis. Ultimately, cytokinesis produces unequal daughter cells, including a big oocyte and a smaller polar body[6].

The main components of the spindle are microtubules that are assembled by polymerized α- and β-tubulin dimers. During prophase I, short and unstable microtubules are scattered throughout the cytoplasm. Chromosomes condense in MI, and then begin to interact with microtubules at many sites. Once the chromosomes are all aligned and associated with microtubules, the microtubules form bipolar arrays that comprise the spindle[7,8].

The regulator of G protein signaling (RGS) proteins negatively regulates G protein signaling[9]. All members of this protein superfamily share a characteristic structure, known as the RGS domain, that exhibits guanosine triphosphatase (GTPase)-activating protein (GAP) activity toward the G protein α subunit, which accelerates the activation of G protein-coupled receptor signaling and affects the deactivation rate[9,10,11]. Although *Rgs2*-deficient mice exhibit normal female fertility[12], it has been shown that *Rgs2* expression can be induced in rat granulosa cells by the administration of human chorionic gonadotropin (hCG)[13], and that the upregulation of RGS2 in human and mouse granulosa cells can inhibit the transcription of Cytochrome c oxidase subunit II (*COX2*), a target gene that is downstream of hCG, via the Gαs pathway[14]. Microarray analysis also identified *Rgs2* as one of the genes regulated by Gonadotropin-releasing hormone (GnRH)[15]. The expression level of RGS2 in human follicular cells has been reported to be associated with the outcome of embryo transfer, suggesting that RGS2 represents a potential biomarker related to the competence of oocyte development and ongoing pregnancies[16,17]. Interestingly, β-tubulin was identified as an RGS2-interacting protein that could directly bind to the N-terminal non-GAP domain of RGS2 and promote microtubule polymerization in vitro in neurons[18]. A recent study reported that RGS2 interacted with Nek-7, which is involved in key events during cell cycle[19], and the interaction between Nek-7 and RGS2 was required for mitotic spindle organization by reducing the amounts of γ-tubulin from the mitotic spindle poles[20]. Additionally, RGS2 affected oocyte maturation by suppressing premature G protein-mediated Ca^2+^ release[21]. Our previous findings also indicated that Rgs2 was distributed on the meiotic spindle of oocytes and that the down-regulation of RGS2 expression mediated by siRNA injection in pronuclear stage embryos resulted in two-cell arrest and delayed embryonic development in mice[22].

Mitogen-activated protein kinase 1/2 (MEK1/2) is an important tyrosine/threonine kinase in the mitogen-activated protein kinase (MAPK)/MEK pathway. Phosphorylated (p)-MEK1/2 plays a important role in cell proliferation and differentiation, as well as the cell cycle, by phosphorylating and regulating down-stream MAPKs and several transcription factors. Previous studies have suggested that MEK is involved in spindle assembly and microtubule dynamics[23]. Additionally, p-MEK1/2 is associated with microtubule organizing centers (MTOCs) and acts either as a microtubule organizing protein or interacts with microtubule organizing proteins to regulate microtubule organization and spindle pole formation during meiosis in oocytes[24].

Because microtubule polymerization and movement are critical processes in meiotic spindle formation and homogenous chromosome separation during oocyte maturation, we hypothesized that the binding of RGS2 and β-tubulin may be required for spindle formation and chromosomal separation in meiosis in oocytes. Thus, in this present study, we examined the expression pattern of RGS2 in mouse oocytes during the first and second meiosis, as well as the co-localization of RGS2 and β-tubulin in meiotic oocytes. We examined meiotic spindle formation and migration during oocyte maturation after inhibiting the binding between RGS2 and β-tubulin using a blocking antibody. Furthermore, p-MEK1/2 was also investigated in oocytes injected with anti-Rgs2 antibody and rabbit IgG at 4 h after GVBD.

## Results

### Localization of Rgs2 protein in oocytes at the MI/II stages and in zygotes

**Fig 1:**
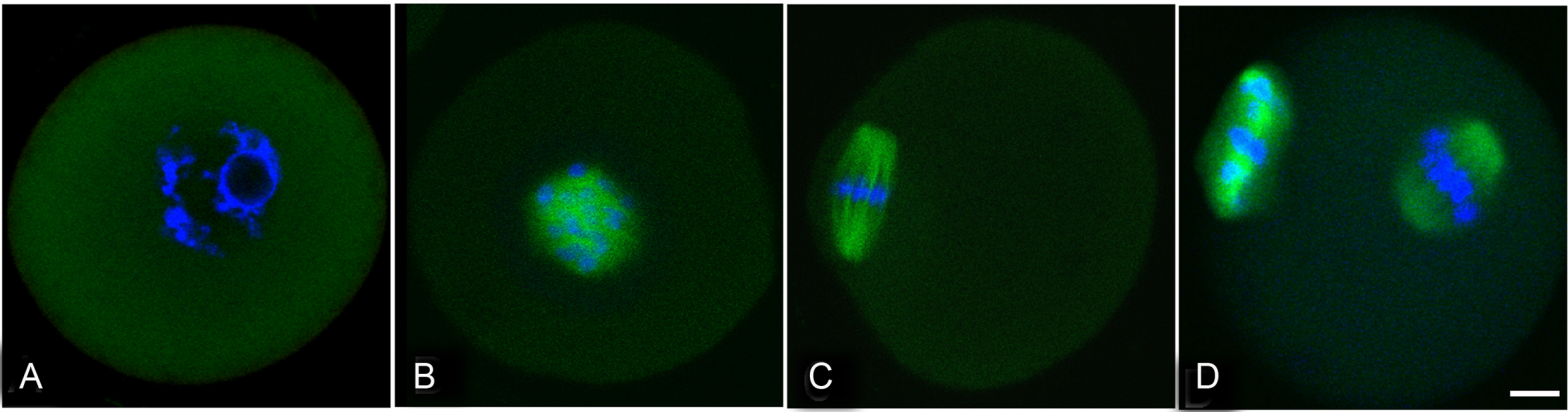
Protein expression of RGS2 in oocytes during maturation in vitro. RGS2 protein expression in mature GV (A), GVBD (B), metaphase I (C), and metaphase II (D) oocytes. RGS2 protein was initially present in the GV ooplasm (A), and 2 h after GVBD it was localized near condensing chromosomes (B). During the first meiotic division, RGS2 accumulated on the spindle (C). In metaphase II oocytes, RGS2 could be observed on the microtubules of spindles and the first polar body (D). Nuclei were stained in blue and RGS2 is shown in green. Scale bar, 20 μm.

**Fig 2:**
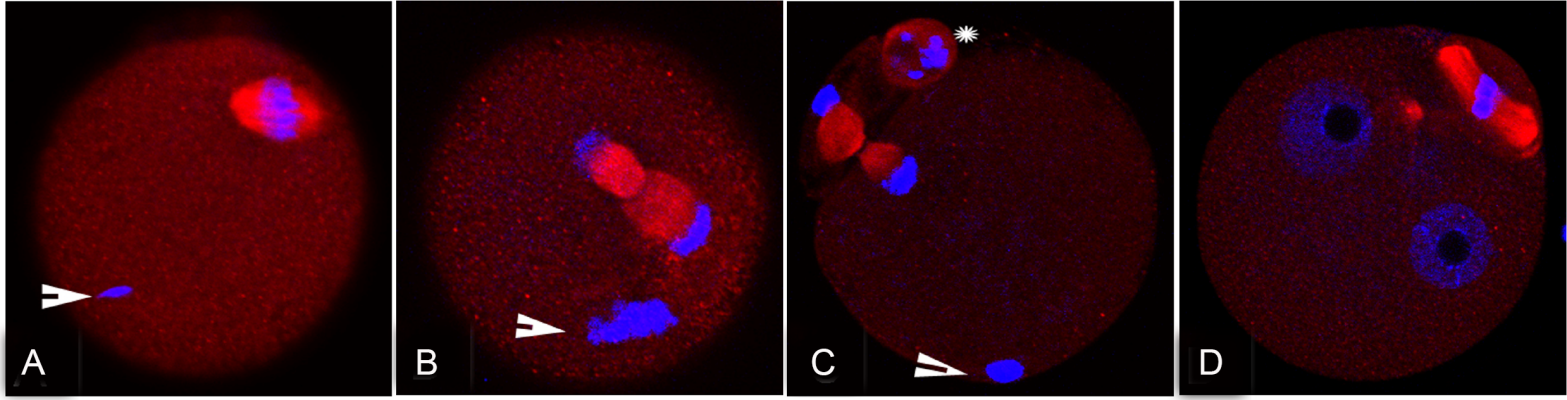
Localization of RGS2 proteins in ICSI oocytes and pronuclear zygotes. RGS2 accumulated on the spindle and sperm DNA (arrow) during meiosis II did not change 30 min after a sperm head was injected into a MII oocyte. By 1 h later, the oocyte had entered the anaphase/telophase stage and RGS2 had become ‘stretched’ to the opposite direction (B). At 2.5 h after ICSI, the second polar body was extruded, and RGS2 was strongly stained in the spindle of fertilized eggs and the second polar body (C). By 5 h after the sperm entered the oocyte, two pronuclei had formed and RGS2 was only present in the zygote cytoplasm and second polar body, but not in pronuclei. Nuclei are stained blue and RGS2 is shown in red. Scale bar, 20 μm.

The localization of Rgs2 in oocytes from the fully-grown GV stage to the MII stage was assessed using immunofluorescent staining. We observed that Rgs2 expression could be detected in oocytes at all stages (Fig 1). In fully-grown GV oocytes, Rgs2 was diffusely distributed throughout the entire cytoplasm, but no signal could be detected in GVs (Fig 2A). The GVBD process in oocytes usually occurs at 2 h of IVM. By 2 h post-GVBD (4 h of IVM), Rgs2 protein began to become concentrated around condensing chromosomes (Fig 1B) and was strongly expressed on the meiotic spindle of MI oocytes (8 h IVM; Fig 1C). At 16 h of IVM, Rgs2 protein markedly accumulated on the meiotic spindle and first polar body of mature MII oocytes (Fig 1D).

To characterize the expression of Rgs2 in oocytes during the process of fertilization, intracytoplasmic sperm injection (ICSI) was performed to obtain fertilized oocyte/eggs. At 0.5, 1.5, 2.5, and 5 h after ICSI, groups of injected oocytes were fixed in 4% PFA for immunofluorescent analysis. The process of polar body extrusion and pronuclear formation has been described in previous studies of human [25] and mouse[26] oocytes. In this present study, we found that RGS2 accumulated on meiosis II spindles, while the sperm nucleus did not begin to decondense at 0.5 h after ICSI (arrow in Fig 2A). After 1 h, oocytes arrested at the anaphase II and telophase II stages; RGS2 protein was distributed on the whole meiotic spindle and moved towards bipolar of spindle, and the chromosomes also started to separate (Fig 2B). At 2.5 h after ICSI, the second polar body was extruded and more abundant Rgs2 protein could be detected on the meiotic spindle and second polar body (Fig 2C). By 5 h after sperm entry, Rgs2 was diffusely distributed throughout the cytoplasm of 2PN zygotes and the intact polar body, but not in pronuclei (Fig 2D).

### Interactions of endogenous Rgs2 with microtubules during oocyte meiosis I

**Fig 3:**
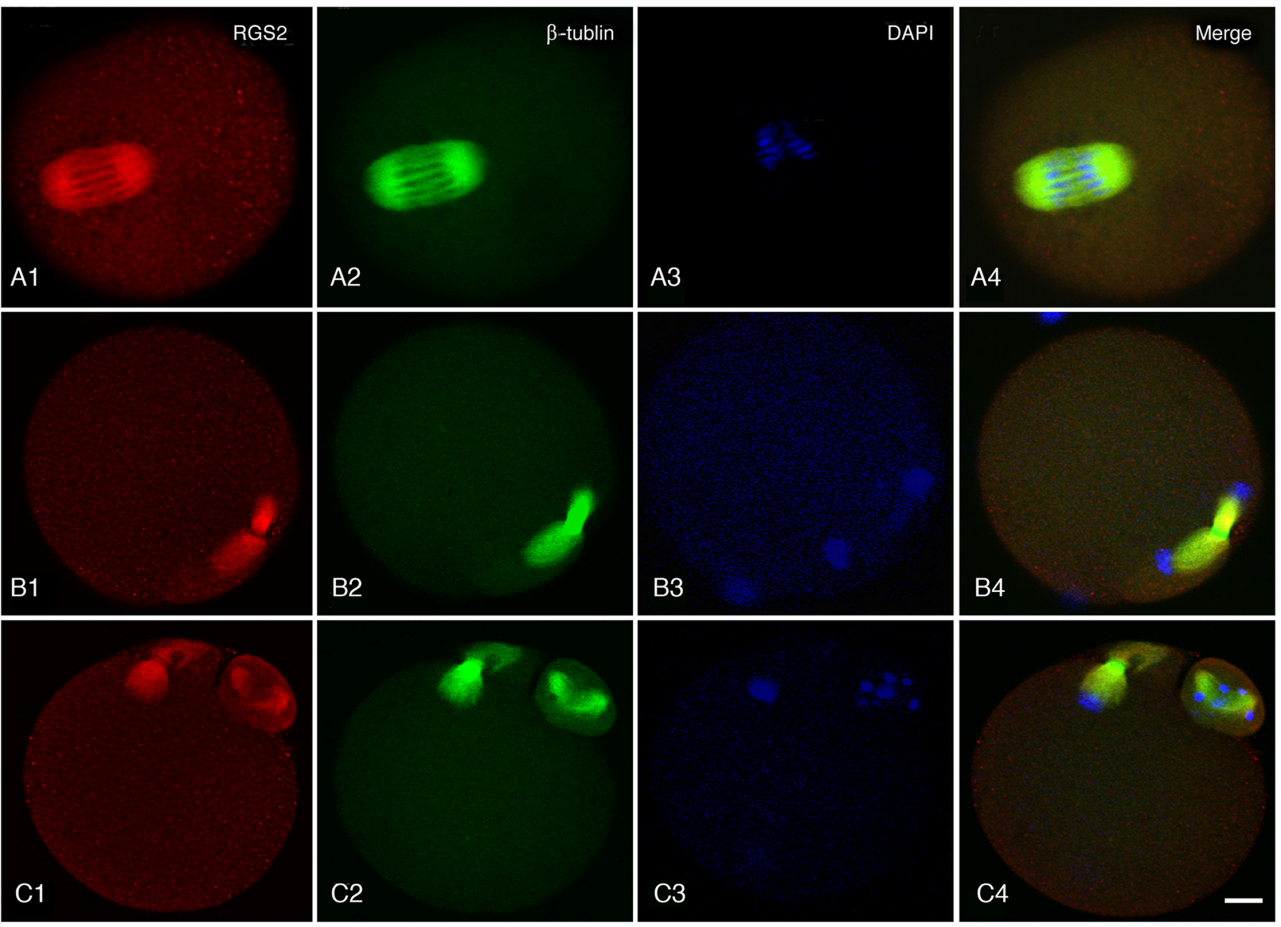
Co-localization of RGS2 and β-tubulin in the meiotic spindle. RGS2 and β-tubulin were co-expressed in spindles of metaphase oocytes (A), anaphase/telophase oocytes (B), and both in telophase oocytes and their polar bodies (C). RGS2 and β-tubulin co-localized on all meiotic spindles. The image shows staining for RGS2 in red, β-tubulin in green, and nuclei in blue. Scale bar, 20 μm.

The subcellular localization of Rgs2 protein in oocytes at different stages of meiosis indicated that Rgs2 likely interacted with microtubules of the meiotic spindle. Therefore, in this present study, immunofluorescent co-staining of Rgs2 and β-tubulin in the spindles of oocytes at meiosis was carried out. Mouse oocytes at three different stages, including metaphase I, anaphase I, and metaphase II, were used to assess the expression of Rgs2 and β-tubulin. We found that Rgs2 and β-tubulin were co-localized in the metaphase spindle (Fig 3A), anaphase spindle (Fig 3B), and polar body (Fig 3C), indicating the potential for an interaction between Rgs2 and β-tubulin to occur during oocyte meiosis I stage.

### Effects of anti-RGS2 antibody on first polar body extrusion of maturing oocytes

To investigate whether interference with the interaction between Rgs2 and β-tubulin could affect oocyte maturation, anti-Rgs2 antibody was injected into GV stage oocytes to block the β-tubulin-binding motif of Rgs2 (i.e., the N-terminal of the Rgs2 protein)[18]. Subsequently, injected oocytes were cultured to estimate the rates of GVBD and first body extrusion. For control oocytes, rabbit IgG was injected into the cytoplasm.

As shown in Table 1, 70.27% and 69.05% of oocytes in the sham-injected and antibody-injected groups, respectively, underwent GVBD to resume the first meiosis; no significant difference existed between these groups. However, a significant difference in first polar body extrusion was detected (73.85% vs. 32.76%), indicating that interference with the binding of Rgs2 to β-tubulin affected first polar body extrusion, but not the resumption of meiosis I.

**Table 1:** Effects of antibody-mediated inhibition of the binding of RGS2 and β-tubulin on GVBD and first polar body extrusion oocyte maturation.

	No. of oocytes	GVBD (%)	1^st^ polar body extrusion (%)
Rgs2 antibody-injected	168	116/185 (69.05^a^)	38/116 (32.76^a^)
Rabbit IgG-injected	185	130/185 (70.27^a^)	96/130 (73.85^b^)

Note: ab, values with different superscript characters within each column indicate a statistically significant difference (P < 0.05). Data were analyzed using the χ^2^ test.

GVBD, germinal vesicle breakdown.

### Effects of anti-Rgs2 antibody injection on meiotic spindle formation and chromosome separation in oocytes

**Fig 4:**
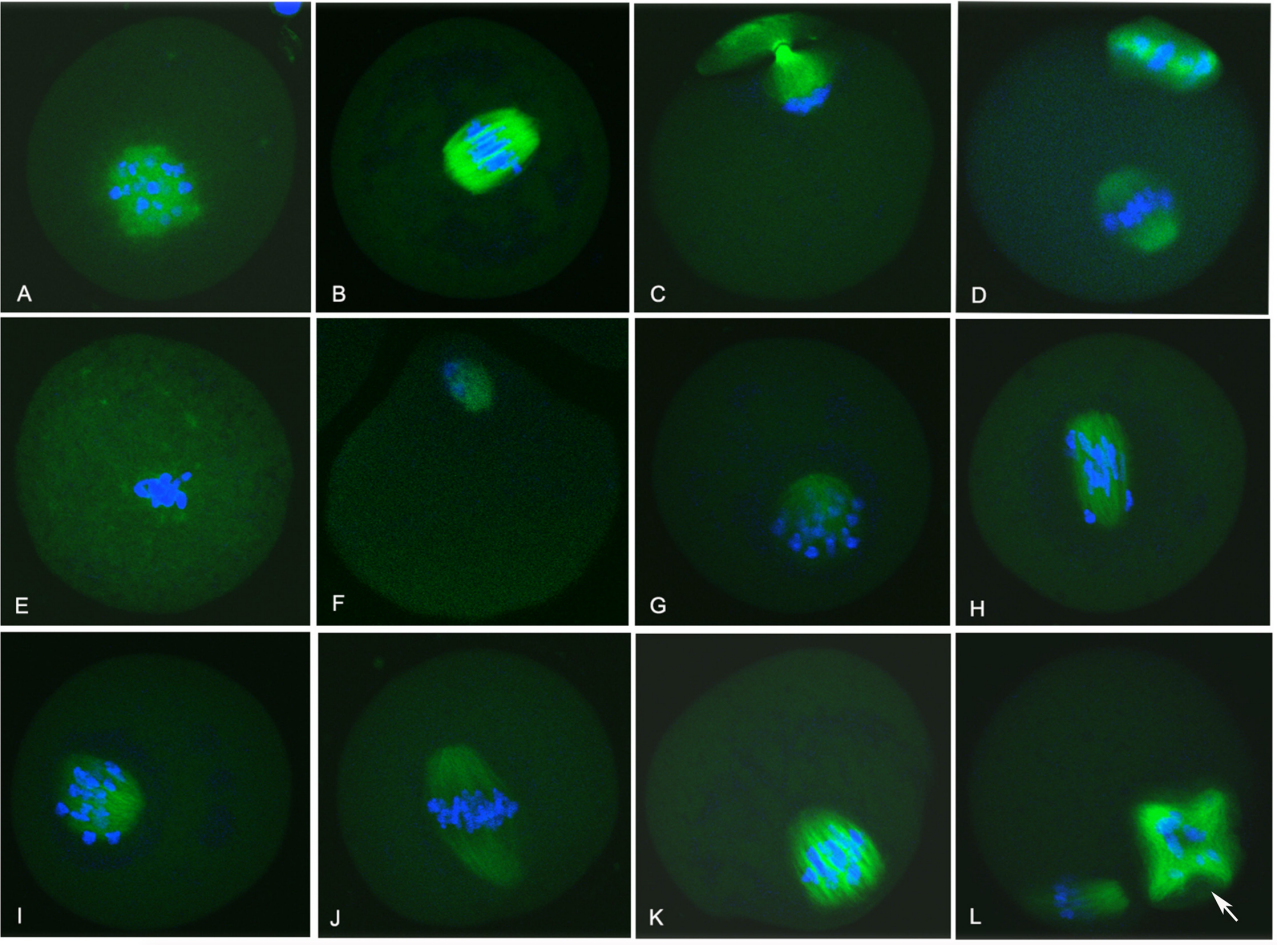
Antibody-mediated inhibition of the binding of RGS2 and β-tubulin in GV oocytes leads to spindle defects during maturation. All stages of control rabbit IgG-injected oocytes were cultured for 1 h (A), 4 h (B), 6 h (C), or 9 h (D) after GVBD, and all oocytes showed normal morphologies. Anti-Rgs2 antibody-injected oocytes were inhibited and exhibited various spindle morphologies (Fig 4E–4L), including microtubule accumulation near the chromatin condensing region, but almost no β-tubulin polymerization in the spindle (Fig 4E). Although a few oocytes were assembled into a normal MI spindle (Fig 4J), most oocytes were blocked at the metaphase I to anaphase I stage (Fig 4F–4K) and were accompanied by spindle defects, such as non-integral spindles (Fig 4G, 4I and 4K) and chromosomal migration failure (Fig 4F, 4H and 4J). Some MII oocytes even extruded first polar bodies that remained morphological abnormal in spindle (Fig 4L, arrow points to a muti-polar spindle). Images show staining of nuclei in blue and RGS2 in green. Scale bar, 20 μm.

To reveal the role of Rgs2 in oocyte meiosis, we further investigated the effects of anti-Rgs2 antibody on meiotic spindle formation in meiotically-arrested oocytes. In the rabbit IgG-injected group, at 4 h of IVM, β-tubulin began to polymerize near the condensing chromatin, and in GVBD oocyte the fluorescent signal became much more intense near the original position of germinal vesicle (Fig 4A). At 8 h of IVM, the spindle was formed and the chromosomes aligned on the center of spindle (Fig 4B). At 10 h of IVM, the spindle microtubule began to drive chromosome segregation (Fig 4C). Then, the first polar body was extruded and oocytes arrested at the MII stage after 16 h of IVM incubation (Fig 4D). In the anti-Rgs2 antibody-injected group, various spindle morphologies could be observed in blocked oocytes (Fig 4E–4L). Several oocytes stopped developing soon after GVBD and microtubules accumulated near the region of chromatin condensation, although less β-tubulin polymerized in the spindle compared with the sham-injected oocytes (Fig 4A and 4E). Although a few oocytes exhibited a normal MI spindle (Fig 4J), most were blocked at the metaphase I to anaphase I stages (Fig 4F–4K) and presented spindle defects, such as non-integral spindles (Fig 4G, 4I and 4K) and chromosome migration failure (Fig 4F, 4H and 4J). For some MII oocytes, although the first polar body was extruded, the morphological abnormal spindle was not bipolarity like a normal oocyte (Fig 4L). This finding indicated that blocking the combination between Rgs2 and β-tubulin could lead to defects in spindle reorganization and chromosome separation in MI oocytes.

### Effects of anti-rgs2 antibody injection on p-MEK1/2 levels in MI oocytes

**Fig 5:**
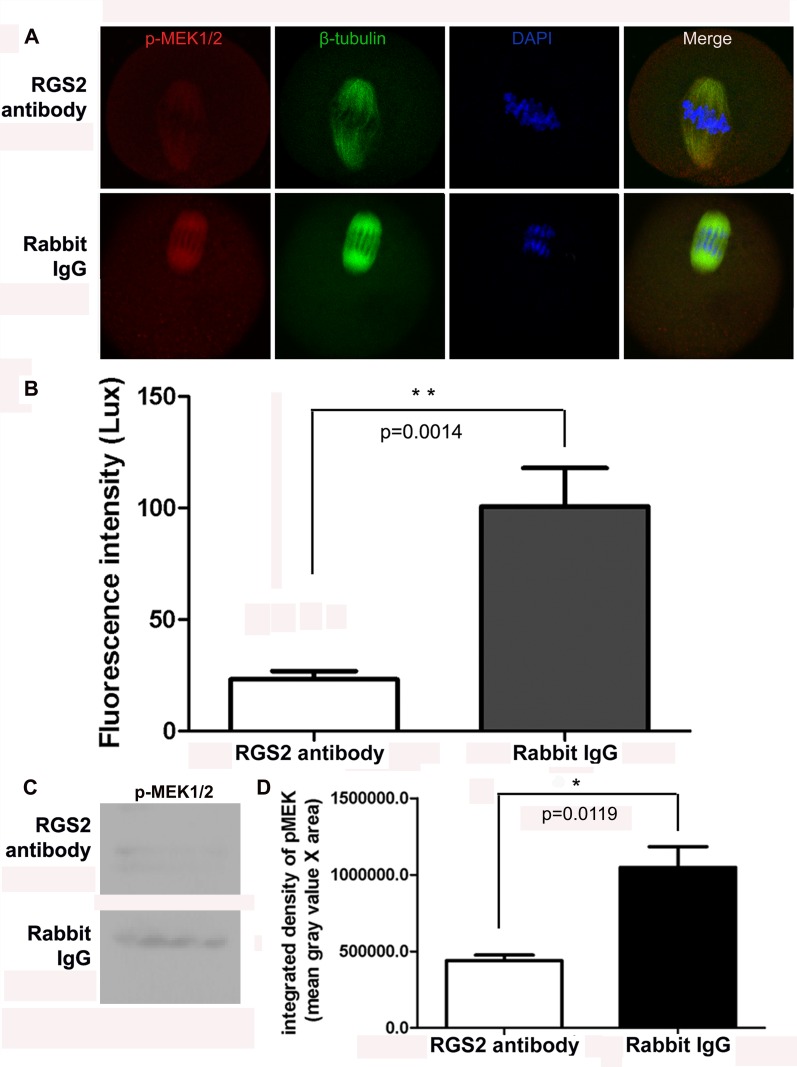
Antibody-mediated inhibition of the binding of RGS2 and β-tubulin reduced the expression of p-MEK1/2 in meiotic spindles during oocyte maturation. (A) p-MEK1/2 was localized in the microtubule-organizing center of spindles in rabbit IgG-injected oocytes. However, in anti-rgs2 antibody-injected oocytes, the p-MEK1/2 signal was very week or not visible. p-MEK1/2 was labeled with Alexa Fluor 594 (red), while β-tubulin was stained with FITC (green) and nuclei were stained by DAPI (blue). (B) Fluorescence intensities of p-MEK1/2 protein in control and anti-Rgs2 antibody-injected oocytes were assessed. Images were obtained using the same laser confocal microscope using the same exposure parameters (HV 520 V; Gain X1; offset 8%). Mean fluorescence intensities were significantly different (*t*-test, p<0.01) between the control IgG- and anti-Rgs2 antibody-injected oocytes; 10 oocytes were measured per group. (C) Western blot analysis of p-MEK1/2 in anti-rgs2 antibody and rabbit IgG-injected oocytes. Blot is representative of 3 independent replicates; each sample includes 50 oocytes. (D) Quantitation of p-MEK1/2 blot. Data are integrated density of blot (mean gray value x blot area) ±SD from three independent experiments. Difference is considered to be significant at p<0.05.

To assess the role of Rgs2 in oocyte meiosis, we also investigated the effects of anti-Rgs2 antibody on MEK1/2 activation. At 4 h after anti-Rgs2 antibody and rabbit IgG injection, a fluorescent signal indicating p-MEK1/2 expression could be observed in the spindle of rabbit IgG-injected oocytes; however, very faint fluorescent signals were detected in oocytes injected with anti-Rgs2 antibody (Fig 5A). The p-MEK1/2 fluorescent signals of 10 oocytes in each group were scanned using a laser confocal microscope with identical exposure parameters (HV 520V; Gain X1; offset 8%) and the mean fluorescence intensity was calculated. We found that inhibition of the binding of Rgs2 and β-tubulin by anti-Rgs2 antibody injection resulted in a significant reduction of p-MEK levels (P<0.01, Fig 5B). Moreover, Western blot assay was performed to explore the effects of *anti-rgs2* antibody injection on p-MEK1/2 level, the results indicated that the expression of p-MEK1/2 protein was significantly lower in antibody injection group compared with the control group (Fig 5C and 5D), which was consistent with fluorescence intensity scanning results.

## Discussion

This present study establishes that Rgs2 can be spatio-temporally expressed in mouse oocytes during the process of in vitro maturation, and that anti-Rgs2 antibody could inhibit oocyte meiosis by inhibiting microtubule polymerization during spindle formation and migration. Antibody-mediated inhibition of Rgs2 could lead to morphological defects in oocyte spindles and extrusion failures of the first polar body, but did not affect the resumption of meiosis I (GVBD).

A previous study showed that Rgs2 was expressed in rat granulosa cells, and its expression could be induced by the administration of hCG[13]. Moreover, levels of Rgs2 expression in granulosa cells were related to the developmental competence of oocytes and ongoing pregnancy in the human mother[16,17]. Nevertheless, the role of Rgs2 during oocyte maturation remains unclear. In our previous study, we found that Rgs2 protein was predominantly expressed in oocytes, granulosa cells, and the corpus luteum of the ovary throughout the estrous cycle in mice; however, Rgs2 expression was not detected in the primordial follicles of premature female mice[22].

In our present study, we found that Rgs2 protein was expressed in cytoplasm of GV oocyte, and highly concentrated on the spindles of oocyte and polar bodies by using immunofluorescent staining. However, the results of Bernhardt et al [21]showed that the RGS2 protein was minimally detected in GV oocytes with western blot assay. We presumed that the difference of Rgs2 protein expression in GV oocyte between two studies was mainly due to the different study methods. Before RGS2 antibody was injected into GV oocytes, RGS2 proteins already existed, therefore the injected RGS2 antibody could only affect the binding of RGS2 protein and β-tubulin, rather than decrease the original level of RGS2 proteins. Rgs2 protein was concentrated and co-localized with β-tubulin in the meiotic spindles. This pattern of co-localization suggested that in addition to being involved in regulating the response of granulosa cells to gonadotropins, Rgs2 might also play an important role in meiosis via effects on microtubule assembly.

The Rgs proteins were initially characterized as inhibitors of signal transduction cascades triggered by G protein-coupled receptors because of their conserved signature RGS domain, which can exhibit activity towards the G protein α subunit[27,28]. In addition to the RGS domain, the NH2-terminal domain has also been described to be an important regulatory site for RGS2[29]. The N-terminus of Rgs2 interacts with adenylylcyclase type III[30], the epithelial Ca^2+^ channel TRPV6[31], and the M1 muscarinic acetylcholine receptor. A previous study established that amino acids 41–60 of the N-terminus of RGS2 can directly bind to β-tubulin, and the association of Rgs2 and β-tubulin might promote microtubule formation by stimulating microtubule polymerization and stabilization in neuronal cells[18]. The major structural elements of the spindle are microtubules, which are composed of two structural tubulin proteins: α- and β-tubulin. These two tubulin proteins form heterodimers during microtubule formation[32]. β-tubulin is a component of the mitotic spindle in multiple cell types[33]. In mouse oocytes, microtubule-organizing centers (MTOCs) act to initiate the meiotic spindle apparatus, and provide a major site of microtubule nucleation[34,35,36]. The protein γ-tubulin is located at the microtubule assembling aster-like structures that surround the chromatin that nucleate microtubules by interacting with α- or β-tubulin monomer subunits at the minus-end of the microtubules at MTOC[37,38]. In this present study, blockade of the binding site of Rgs2 and β-tubulin using an anti-Rgs2 antibody injected into GV oocytes resulted in the failure of cytokinesis and second polar body extrusion during the process of in vitro maturation. This observation suggested that Rgs2 might play an important role in oocyte maturation by binding to β-tubulin. Moreover, immunoflourescent staining for β-tubulin also indicated that the spindles of MI blocked-oocytes exhibited obvious morphological defects and could not properly attract chromosomes to the two poles. Previous studies showed that Rgs2 could act as a Nek-7 interactor to affect the function of γ-tubulin, a highly conserved component of MTOCs, and reported that Rgs2 depletion led to mitotic delay and severe defects in chromosome alignment and congression [19,20]. A recent study [21] found that RGS2 depletion by RNAi did not affect oocyte maturation to MII stage, and the author inferred that RGS2 was not required during oocyte maturation. However, in present study we directly blocked the binding site of Rgs2 protein and β-tubulin, and the binding inhibition of two proteins resulted in the significant reduction of first polar body extrusion and the abnormal spindle formation. In RGS gene family, beside RGS2 gene there are many other members such as RGS12 or RGS14 [39], which could express in mouse germinal vesicle oocyte, metaphase II oocyte or zygotes. When RGS2 was suppressed or deleted at gene levels, these subfamily members might substitute of RGS2 gene to function as a compensation through other signaling pathways, therefore it possibly appearred that RGS2 gene was deleted or suppressed, but oocyte maturation apart from the litter-szie was not affected [21]. However, in the present study we inject a RGS2-specific antibody to directly inhibit the binding of RGS2 protein and beta-tubulin, rather than interfere or delete RGS2 at gene or protein expression levels. Moreover, we eventually found that this inhibition of the binding between RGS2 and beta-tubulin could affect spindle formation and chromosome segregation of oocytes.

It is well established that MEK1/2 is involved in controlling spindle assembly and microtubule dynamics[23], and that p-MEK1/2 acts as a microtubule organizing protein or interacts with microtubule organizing proteins to regulate microtubule organization and spindle pole formation during meiosis in mouse oocytes[24]. Various studies have shown that MEK1/2 is involved in regulating microtubule assembly and spindle organization by interacting with polo-like kinase 1[40], regulating formin activity, or mediating polo-like phosphorylation of FAK on Serine 732[41]. Our present findings indicate that inhibition of the binding sites of Rgs2 and β-tubulin with an anti-Rgs2 antibody significantly reduced the levels of p-MEK on the meiotic spindle, suggesting that Rgs2 accelerates MEK inactivation during microtubule assembly.

In summary, we demonstrated herein that Rgs2 protein was expressed and localized in meiotic spindles of mouse oocytes from the resumption of the first meiosis to pronuclei formation in vitro. Blockade of the binding sites between Rgs2 and β-tubulin with an anti-Rgs2 antibody in GV oocytes resulted in defective meiotic spindle reorganization, failed chromosomal segregation, and reduced p-MEK1/2 expression during IVM, suggesting that RGS2 might be involved in oocyte maturation by regulating the polymerization of β-tubulin-containing microtubules and MEK1/2 activation during spindle formation and chromosome separation.

## Materials and Methods

### Use and ethics statement of animals, and sample preparation

A total of 100 B6D2F1 (C57BL/6 × DBA/2) female mice of 3 or 8–10 weeks of age were used in this present study. All mice were purchased from the Guangzhou Animal Center (Guangzhou, China), and were housed at a controlled temperature (~22°C) under a 14 h light: 10 h dark photoperiod. Mouse health status was monitored once daily. All mice used in this study were physically normal and healthy. No any disease and death occurred in these mice. All experiments were carried out in full compliance with standard laboratory animal care protocols and were approved by the Ethical Committee of the Guangdong NO.2 Provincial People’s Hospital (No. 2014-KYLLM-065.

### Superovulation and oocyte collection

B6D2F1 (C57BL/6 × DBA/2) female mice that were 3 weeks of age were superovulated by injecting 7.5 IU pregnant mare serum gonadotrophin (PMSG; Tianjin Animal Hormone Factory, Tianjin City, China) and sacrificed by cervical dislocation for ovarian tissue collection 48 h later. The collected ovarian tissues were placed directly into pre-warmed (37°C) M2 medium (Sigma–Aldrich, St. Louis, MO, USA) supplemented with 4 mg/ml bovine serum albumin (Sigma–Aldrich); 50 μg/ml dibutiryl cyclic AMP (dbc AMP; Sigma–Aldrich) was added to inhibit the resumption of meiotic maturation. Ovarian follicles were punctured with a 27-gauge needle to release the enclosed oocytes. Germinal vesicle (GV) oocytes were collected and cultured further in M2 medium supplemented with 50 μg/ml dbcAMP under embryo-tested light mineral oil (Sigma–Aldrich) at 37°C in a 5% CO_2_ atmosphere following IVM and antibody injection.

For oocyte collection using in intracytoplasmic sperm injection (ICSI), adult female mice that were 12-weeks-old were injected with 7.5 IU PMSG, followed by 7.5 IU human chorionic gonadotrophin (hCG; Ningbo Animal Hormone Factory, Ningbo City, China) 46–48 h later. Mice were killed by cervical dislocation and oocytes were then collected from the oviducts 13–15 h after hCG injection. After oocyte collection, cumulus cells were dispersed with 0.1% hyaluronidase in droplets of HEPES-buffered CZB medium (HEPES-CZB). After several minutes, oocytes were transferred to fresh droplets of HEPES-CZB and were denuded of almost all cumulus cells by gentle pipetting. Denuded oocytes that exhibited a homogeneous ooplasm were selected and re-suspended in fresh droplets of potassium simplex optimized medium (KSOMaa, Millipore), which had been previously covered with paraffin oil (Sigma). Oocytes were cultured at 37°C in a 5% CO_2_ atmosphere until further use. Fresh spermatozoa were collected from the epididymes of a mature B6D2F1 male mouse for each experiment and were incubated in KSOMaa for 30 min at 37°C in a 5% CO_2_ atmosphere.

### Intracytoplasmic sperm injection (ICSI)

ICSI was performed as described previously[42]. A Leica Hofman microscope (LSM6000) equipped with a TransferMan NK2 micromanipulator and a piezo-driven unit (Eppendorf, Germany) was used. Briefly, spermatozoa were washed three times with HEPES-CZB and 1 μL aliquots of sperm solution were placed in droplets (~10 μL) of HEPES-CZB that contained 10% polyvinylpyrolidone (Sigma–Aldrich) in a micromanipulation chamber. After washing, sperm heads were separated from the tail by subjecting the head–tail junction to several piezo pulses. After sperm injection, oocytes were cultured in KSOMaa medium at 37°C in a 5% CO_2_ atmosphere. Oocytes were fixed at 0.5, 1.5, 2.5, and 5 h for analysis by immunofluorescence.

### *In vitro* maturation (IVM) and collection of oocytes at different stages

For IVM, GV oocytes were obtained from 12-week-old mice that had been superovulated by injecting 7.5 IU PMSG per mouse for 48 h. Cumulus-oocyte complexes (COCs) were directly collected from ovaries by puncturing the follicles under a stereomicroscope with 27-gauge needles. All collected GV oocytes were placed in 50 μl microdrops of alpha-Minimum Essential Medium Eagle (α-MEM) supplemented with 5% fetal bovine serum (FBS) and incubated in a humidified atmosphere of 5% CO_2_ at 37°C for 14–16 h. Oocytes were collected after 0, 4, 8 and 16 h of incubation. At the same time, oocytes were observed at regular intervals using an inverted microscope to assess the development stages of oocytes based on morphological changes in the nuclei or extrusion of the first polar body.

### Microinjection of anti-RGS2 antibody

Microinjection of anti-RGS2 antibody was performed in M2 medium using a TransferMan NK2 micromanipulator equipped with a FemtoJet microinjector (Eppendorf, Germany). A total of 5–10 pl of rabbit anti-RGS2 polyclonal antibody (Santa Cruz Biotech, 1:2 dilution to 0.2 mg/ml) was injected into the cytoplasm of each GV oocyte. The same volume of rabbit IgG solution (0.2 mg/ml; Protein Tech Group, Chicago, IL, USA) was injected as a control. For each batch of injections, ~50 oocytes were injected, and each treatment was repeated at least three times. After injections, oocytes were incubated in α-MEM with FBS at 37°C in 5% CO_2_ atmosphere following the aforementioned IVM procedure. Oocytes were collected 4, 8, 10, and 16 h after IVM for analysis by immunofluorescence.

### Immunofluorescent staining and western blot assay

Immunofluorescent staining was carried out as described previously[22]. Briefly, all oocytes and embryos were fixed with 4% (w/v) paraformaldehyde in PBS for 40 min at room temperature, and then were permeabilized in PBS containing 0.1% (w/v) Triton X-100 and 0.3% BSA for 30–40 min at 37°C. After washing twice with PBS containing 0.01% Triton X-100, oocytes were incubated in blocking solution (PBS containing 150 mM glycine and 0.3% BSA) for 30 min at 37°C. Samples used for RGS2 expression pattern analysis were incubated with rabbit anti-mouse RGS2 antibody (Abcam, Cambridge, MA, USA), β-tubulin (Abcam), or pERK1/2 (Abcam) that had been diluted (1:200) in blocking solution for 40 min at 37°C or overnight at 4–8°C, and then were incubated with Alexa Fluor 488 (green) or Alexa Fluor 594 (red) conjugated donkey anti-rabbit IgG (H+L) antibody (Invitrogen/Life Technologies, Carlsbad, CA, USA) at a 1:2000 dilution in blocking buffer for 30 min at 37°C. Samples used for β-tubulin analysis were incubated with anti-β-tubulin-FITC antibody (Sigma–Aldrich) for 30 min at 37°C. Finally, all samples were mounted on slides with anti-fluorescence fade medium (DABCO, 1,4-diazabicyclo [2.2.2] octane). Embryos used for subcellular localization analysis were examined with a laser-scanning confocal microscope (Zeiss LSM 510 Meta, Jena, Germany). Images shown in the results section are representatives of at least 80 samples from more than 3 replicates. All images were captured under a 40X objective (magnification 630; resolution 1300X1030; pixels at 150 pixels per inch) using the same exposure parameters (HV 520 V; Gain X1; offset 8%) for all images. Intensity of fluorescence was measured with Image J software (National Institutes of Health, Bethesda, MD, USA). The mean value of fluorescence intensity for each oocyte was corrected by subtracting the mean fluorescence of the negative control.

For western blot analysis, the whole protein of 50-oocyte/sample were extracted in lysate buffer (50mM HEPES, 150mM NaCl, 1mM EGTA, 1.5mM MgCl2, 100mM NaF, 10% glycerol and 1% Triton X-100, 1Mm PMSF, 10ug/ml aprotinin and 1mM sodium orthovanadate) and then boiled in 20ul loading buffer (100mM Tris, pH 6.8, 4% SDS, 20% glycerol, 10% β-mercaptoethanol, 0.2% bromophenol blue), fractionated by electrophoresis in 12% SDS polyacrylaminde gels, and transferred to nitrocellulose membranes (Invitrogen). Blots were blocked in 5% BSA solution, and then incubated overnight at 4°C with antibody against pERK1/2 (Abcam) at 1:800 dilution in blocking buffer, and HRP-conjugated goat anti-rabbit secondary antibody (Santa Cruz) at 1:3000 dilution in TBST for 1hour. Color development was performed using the Enhance chemiluminescence system (Pierce, Rockford, IL, USA). Quantitation of p-MEK1/2 blot was performed and the data are integrated density of blot (mean gray value x blot area) ±SD from three independent experiments.

### Statistical analysis

The rates of GVBD and first body extrusion in anti-RGS2 antibody- and rabbit IgG-injected oocytes were evaluated using the chi-square (χ^2^) test. To compare the fluorescence intensities of p-MEK1/2 protein between the anti-RGS2 antibody- and rabbit IgG-injected oocyte groups, Student’s *t*-test was used. A threshold for statistical significance was set at P<0.05.
